# Effectiveness of antioxidants (propolis, blueberry, vitamin E) associated with verapamil in the medical management of Peyronie's disease: a study of 151 cases

**DOI:** 10.1111/j.1365-2605.2011.01219.x

**Published:** 2012-08

**Authors:** G Paulis, R D'Ascenzo, P Nupieri, G De Giorgio, G Orsolini, T Brancato, R Alvaro

**Affiliations:** * Andrology Service, Regina Apostolorum HospitalAlbano Laziale (Rome, Italy); † Complex Operative Unit of UrologyRome, Italy; ‡ Jewish HospitalRome, Italy; § Department of Public Health and Cellular Biology, University of Rome “Tor Vergata”Rome, Italy

**Keywords:** anthocyanides, iontophoresis, Peyronie's disease, propolis, verapamil, vitamin E

## Abstract

A total of 151 patients (age: 24–74 years, mean: 55 ± 10.3) diagnosed with Peyronie's disease were enrolled in a non-surgical treatment. In addition to medical histories and physical examinations, all patients underwent the following tests: penile ultrasound, IIEF questionnaire and photographic documentation. The penile curvature was measured by taking a photograph during maximum erection. All 151 patients were treated at different times and with different combinations of drugs, and afterwards, they were clinically studied and divided into five different treatment groups: 1st = verapamil (injection + iontophoresis) + vitamin E + topical diclofenac + blueberries; 2nd = verapamil (injection + iontophoresis) + vitamin E + topical diclofenac + propolis; 3rd = verapamil (injection) + vitamin E + topical Diclofenac; 4th = verapamil (iontophoresis) + vitamin E + topical diclofenac; 5th = verapamil (injection + iontophoresis) + topical diclofenac + blueberries + propolis. All patients were treated for 6 months after which they underwent the same follow-up tests as performed prior to the treatment. The following was achieved: group 1 had the most reduction in plaque size (−66.4%; *p* = 0.000), group 2 obtained the highest rate where penile curvature disappeared (24.5%; *p* = 0.019); the best results with reference to decrease in curvature angle were reached by the 2nd group (**−**14°) and group 1 obtained **−**9.6° (*p* = 0.000).

## Introduction

Peyronie's disease (PD) is a localized connective tissue disorder characterized by a fibrous inelastic plaque involving the tunica albuginea of the penis. Possible signs of this disease are: penile pain, curvature, difficulty with coitus and penile deformity. The aetiology remains unknown, although in recent years pathophysiological knowledge has evolved, and new studies propose that penile trauma was the cause of the disease (Devine *et al.*, 1997; Zargooshi, 2004). Recent studies indicate a prevalence of 3.2–8.9% in adult men (Schwarzer *et al.*, 2001; Mulhall *et al.*, 2004). In his research, SIMONA Group Study showed a significant correlation between cigarette smoking and PD (La Pera *et al.*, 2001). Although some studies proposed the spontaneous resolution in patients who received no treatment (Williams & Thomas, 1970; Gelbard *et al.*, 1990), a research study (Mulhall *et al.*, 2006) has achieved a worsening of penile curvature in 48% of cases at follow-up.

In the course of the inflammatory process, PD occurs as a result of the activation of nuclear factor kappa B (NF-κB), which induces the expression of iNOS with an increase of nitric oxide (NO) that is incompatible with SOD, leading to the increased production of peroxynitrite anion (ONOO^−^) (Sikka & Hellstrom, 2002). All these processes result in the activation and differentiation of fibroblasts and overproduction of collagen (fibrous plaque).

Conservative medical treatment is indicated in the development stage of the disease, for at least 1 year after diagnosis and whenever there is penile pain. Current non-surgical therapies with varying success include: para-aminobenzoate, vitamin E, colchicine, tamoxifen, propoleum (propolis), verapamil, interferons, collagenase, cortisone, pentoxifylline, superoxide dismutate, iontophoresis, radiation and ESWT (Hauck *et al.*, 2006; Trost *et al.*, 2007; Taylor & Levine, 2008; Safarinejad *et al.*, 2010). Noting the extreme variability of results of the different conservative treatments proposed in international literature, the objective was to carry out a retrospective study on a considerable number of patients with PD treated in association with several drugs –‘combination therapy’– at the hospital over the past 11 years. In particular, the effectiveness of verapamil (injection and/or iontophoresis) and several antioxidants: vitamin E, blueberries (anthocyanins) and propolis was investigated.

*Verapamil* is a slow calcium channel blocker that reduces the local production of extracellular matrixes (by fibroblasts) and the proliferation of fibroblasts. Verapamil increases the local activity of collagenase and affects the cytokine regulation of fibroblasts reducing the excess production of fibrogenic cytokines (Levine *et al.*, 1994; Roth *et al.*, 1996; Levine & Estrada, 2002; Mulhall *et al.*, 2002).

*Iontophoresis* (electromotive drug administration/EMDA) is a non-invasive treatment for drug administration without the use of injections and the patient can autonomously perform this therapy. Levine *et al.* (2003) who measured the levels of verapamil in the tunica albuginea in patients, after surgical treatment of PD, showed that the verapamil was detected in 71.5% of the tunica albuginea specimens after iontophoresis administration with a wide range of drug levels. *Antioxidants* may play an important role in medical treatment of PD because the accumulation of activated inflammatory cells in PD leads to the production of ROS (Sikka & Hellstrom, 2002).

*Vitamin E* is a potent antioxidant. Some studies have found that vitamin E and its metabolites have an anti-inflammatory and anti-COX2 property (O'Leary *et al.*, 2004; Jang *et al.*, 2008). Hauck *et al.* (2006) argued that, despite vitamin E being widely used, there is no evidence that it has a significant effect on the symptoms of PD. Possible toxicity caused by chronic intake of vitamin E has often been discussed in medical literature; however, regarding the toxicity–mortality rate as a result of the accumulation of vitamin E, the latest meta-analysis has shown that this event does not seem to occur with doses up to 5500 IU/daily (Abner *et al.*, 2011). Concerning the conversion between *mg* and *IU* in the dosage of vitamin E, see the *note* at the end of Materials & methods.

*Propolis* is a resinous mixture that honey bees extract from the buds or sap of trees and other plants. Honey bees use it to seal the small open spaces in their hives and to protect themselves from the cold, wind and rain and from attacks by other insects. The most interesting property of propolis is to prevent disease by protecting the colony of bees from parasites, bacteria and other microbes. The major components of this substance are: caffeic acid phenethyl ester (CAPE), phenolics, terpenes, hydrocarbons, aromatic and aliphatic acids, flavonoids (Pinocembrin, Galangin, Chrysin etc.) (Schnitzler *et al.*, 2010). Propolis has anti-inflammatory and antioxidant properties (Russo *et al.*, 2002) and, in particular, inhibits NF-κB (because of its component CAPE) and the production of interleukins (Natarajan *et al.*, 1996; Marquez *et al.*, 2004; Ha *et al.*, 2010).

*Blueberries* are flowering plants belonging to *Vaccinium* spp. (*Ericaceae*). Several authors have indicated that this fruit contain anthocyanins, polyphenols and flavonoids and appear to have the highest antioxidant capacity among common fruits and vegetables (Prior *et al.*, 2000; Wu *et al.*, 2004); blueberries may also cure chronic diseases through anti-inflammatory and anti-fibrotic effect mechanisms (Boniface & Robert, 1996; Choi *et al.*, 2010). Blueberry anthocyanins are also able to inhibit NF-κB, iNOS and COX-2 expression (Karlsen *et al.*, 2007; Wang *et al.*, 2008).

*Diclofenac:* the properties of NSAID's, including the anti-inflammatory activity of diclofenac, are already known.

## Materials & methods

This is a retrospective study. From 1999 to December 2010, 151 patients with Peyronie's disease were enrolled for a non-surgical treatment. From the original 207 cases, for various reasons, 56 cases were excluded from the treatment: two patients with advanced age (potential negative effects of verapamil on atrioventricular conduction); three patients with cardiac disease or patients with a pacemaker; eight patients with stabilized Peyronie's disease; 43 patients who did not complete diagnostic tests, therapy or follow-up.

All 151 patients were treated at different times and with different combinations of drugs and were then studied and divided into five groups:

1. Verapamil Injection (peri-lesional) 10 mg/every 2 weeks (12 total injections – the first injection only 5 mg) + iontophoresis with 5 mg daily, excluding the day of injection) + vitamin E 600 mg/oral/daily + topical diclofenac sodium 4% gel/twice a day + blueberries 160 mg/oral/daily – for 6 months; 33 patients treated from 1999 to 2004;
2. Verapamil Injection (peri-lesional) 10 mg/every 2 weeks (12 total injections – the first injection only 5 mg) + iontophoresis with 5 mg daily, excluding the day of injection) + vitamin E 600 mg/oral/daily + topical diclofenac sodium 4% gel/twice a day + propolis 600 mg/oral/daily (on an empty stomach to facilitate the intestinal absorption) – for 6 months; 65 patients treated from 2005 to March 2009;
3. Verapamil Injection (peri-lesional) 10 mg/every 2 weeks (12 total injections – the first injection only 5 mg) + vitamin E 600 mg/oral/daily + topical diclofenac sodium 4% gel/twice a day – for 6 months; 13 patients who expressed a desire not to be subjected to iontophoresis with verapamil;
4. Verapamil (5 mg/daily) by iontophoresis + vitamin E 600 mg/oral/daily + topical diclofenac sodium 4% gel/twice a day – for 6 months; 14 patients who expressed a desire not to be subjected to perilesional injections with verapamil;
5. Verapamil injection (peri-lesional) 10 mg/every 2 weeks (12 total injections – the first injection only 5 mg) + iontophoresis with 5 mg daily (excluding the day of injection) + topical diclofenac sodium 4% gel/twice a day + blueberries 160 mg/oral/daily + propolis 600 mg/oral/daily (on an empty stomach to facilitate the intestinal absorption) – for 6 months; 26 patients treated from April, 2009 to December, 2010.

None of the 151 patients have taken PDE5 inhibitors during the study. In fact, 19 patients who had practiced PDE5 inhibitors’ therapy were initially excluded from the study. After which, all of them were admitted to this study, but only after having discontinued the drug for at least a month.

Although this is a retrospective study, it is interesting to note that the different treatment groups are sufficiently homogeneous (patient age, living habits, presence or absence of penile pain, plaque volume, degree of curvature, presence or absence of erectile dysfunction) for a possible statistical analysis of results.

To avoid iatrogenic episodes of hypotension or disorders in A-V conduction in the case of a hypertensive patient treated with calcium channel blockers, it was suggested that the patient not take the daily dose of such drugs on the day of the perilesional injection. When iontophoresis was chosen as an additional method of administration (groups: 1, 2, 4, 5), equipment produced by the company Androline (Milan, Italy) was used placing the drug verapamil (5 mg) on the positive pole with 4 mA output and for a duration of 20 min/day. The blueberries administered to groups 1 and 5 were a pharmaceutical preparation containing blueberry extract (36% anthocyanosides). Vitamin E was prescribed to patients in groups 1, 2, 3 and 4, and is a pharmaceutical preparation in the form of dl-α-tocopherol acetate (see note at the end of this paragraph). Having considered the conclusions made by Hauck *et al.* (2006) and other authors (Hashimoto *et al.*, 2006; Trost *et al.*, 2007) regarding the results (inefficacy) of the use of vitamin E therapy in patients with Peyronie's disease, from April 2009, vitamin E has been excluded from the ‘combination therapy’ (group 5) combining blueberries and propolis instead, which have showed good efficacy – in this experience and that of Lemourt Oliva *et al.* (1998, 2003a, b, 2005)– in treating the disease by reducing plaque size and penile curvature.

*Main outcome measures:* In addition to medical history and physical examinations, all patients underwent the following tests: penile ultrasound, penile X-ray (only in case of detection of a ‘shadow-cone’ during the ultrasound examination), IIEF questionnaire (International Index of Erectile Function) (Rosen *et al.*, 1997) and the penile curvature that was measured by taking a photograph during maximum erection. All patients were evaluated with the IIEF. The total of the scores relating to questions 1–5 and 15 of the IIEF domain was calculated, with a maximum score of 30. Patients who had a total score of less than 26 were identified as having erectile dysfunction. When statistical analysis was performed after treatment, significant improvement in erectile function occurred when the total IIEF score was increased by at least 6 points.

All patients were treated for a total time of 6 months and then underwent the same tests as performed prior to the treatment.

A statistically significant consideration was when the *p*-value was less than 0.05. The results of this study were subject to statistical analysis using primer of biostatistics (by Stanton A. Glantz) software. This work was carried out in accordance with the Helsinki Declaration of 1975 as revised in 1983.

Note:

Conversion between mg and IU in the dosage of vitamin E (Eitenmiller & Lee, 2004):

IU = unit of measurement that expresses the quantity of active vitamin E

dl-α-tocopherol acetate (synthetic ester of vitamin E) 1 mg = 1 IU

dl-α-tocopherol succinate (synthetic ester of vitamin E) 1 mg = 0.89 IU

dl-α-tocopherol (synthetic vitamin E) 1 mg = 1.10 IU

d-α-tocopherol acetate (natural ester of vitamin E) 1 mg = 1.36 IU

d-α-tocopherol succinate (natural ester of vitamin E) 1 mg = 1.21 IU

d-α-tocopherol (natural vitamin E) 1 mg = 1.49 IU

*For those interested in finding correspondence between the chemical form of vitamin E and the commercial name of the drug, please consult the pharmaceutical database on Internet:*http://drugs-about.com/ing/vitamin-e.html

## Results

The study involved 151 patients (mean age = 55.3 ± 10.3 years – range 24–74 years). In most cases (93.37%), patients reported a penile curvature (141/151 cases) and the penile curvature angle was between 10° and 45° in 91.48% of the cases (129/141). Penile pain was present in 80 patients (52.98%). Erectile dysfunction was present in 52 cases (34.4%).

The location of the plaques was as follows: basal third of the penis = 22 patients (14.5%); between the basal and middle third of the penis = six cases (3.9%); middle third of the penis = 37 cases (24.5%); between the middle and distal third of the penis = 30 cases (19.8%); distal third of the penis = 45 cases (29.8%); multiple sites of the penis = 11 cases (7.2%) of which in seven cases in the basal third + another plaque in the middle third of the penis and in four cases in the basal third + another plaque in the distal third of the penis.

In 112 patients (74.17%), the site of the penile plaque was found in the middle or distal third of the penis. In most cases (54.3%), the penile plaques were hyperechoic (82 cases) (after a *penile ultrasound examination*), in 17 cases (11.2%) the plaques were hypoechoic, in six cases (3.97%) the plaques were isoechoic and in the rest of the cases (30.46%), the plaques resulted heterogeneous (46 cases). In most cases (64.9%), the plaque size was found to be between 1 mm and 20 mm (98 cases). Relating to the 82 cases of hyperechoic penile plaques, in 70 of these cases, the medical report of the *penile ultrasound examination* had pointed out a ‘shadow-cone’ with the meaning of calcification. Afterwards, all 70 patients underwent a *penile X-ray*, after which only in 44 patients (44/70 = 62.8%) (*p* = 0.000 *McNemar Test*), the hyperechoic plaque was found partially or completely calcified. After treatment, different results were found on the basis of the echogenicity of the penile plaques (see below).

After completing 6 months of various types of ‘combination therapy’ and after the programmed follow-up, the results were detected with statistical analysis (see the Table 1 of results)

**Table 1:** Table of results (post-treatment)

	Group 1 (%)	Group 2 (%)	Group 3 (%)	Group 4 (%)	Group 5 (%)	Statistical analysis (*p-*value)
Pain disappearance (*n* patients/total patients)	100 (10/10)	90.0 (36/40)	83.3 (5/6)	88.8 (8/9)	80.0 (12/15)	0.617 (χ^2^ test)
Success in reducing the plaque size (*n* patients/total patients)	90.9 (30/33)	76.9 (50/65)	84.6 (11/13)	85.7 (12/14)	73.0 (19/26)	0.387 (χ^2^ test)
Effective reduction in plaque size (mean rate % + standard deviation)	−66.43 ± 29.43	−50.01 ± 34.75	−41.56 ± 21.68	−32.50 ± 12.88	−33.2 ± 26.8	0.000 (anova)
Disappearance of the plaque (*n* patients/total patients)	27.2 (9/33)	7.69 (5/65)	0 (0/13)	0 (0/14)	0 (0/26)	0.001 (χ^2^ test)
Increase of plaque size (*n* patients/total patients)	3.0 (1/33)	3.0 (2/65)	15.3 (2/13)	14.2 (2/14)	7.69 (2/26)	0.249 (χ^2^ test)
Success in reducing the penile calcification (*n* patients/total patients)	100 (10/10)	92.0 (23/25)	50 (1/2)	100 (1/1)	50 (3/6)	0.022 (χ^2^ test)
Effective reduction in penile calcification size (mean rate % + standard deviation)	−60.9 ± 34.37	−49.6 ± 29.7	−10.0 ± 7.07	−50.0 ± 0	−51.6 ± 41.93	0.257 (anova)
Disappearance of the penile calcification (*n* patients/total patients)	30.0 (3/10)	20.0 (5/25)	50 (1/2)	100 (1/1)	16.6 (1/6)	0.360 (χ^2^ test)
Improvement of penile curvature (*n* patients/total patients)	74.1 (23/31)	80.3 (49/61)	90.9 (10/11)	69.2 (9/13)	52.0 (13/25)	0.054 (χ^2^ test)
Disappearance of the penile curvature *n* patients/total patients	3.2 (1/31)	24.5 (15/61)	9.0 (1/11)	7.7 (1/13)	4.0 (1/25)	0.019 (χ^2^ test)
Worsening of penile curvature (*n* patients/total patients)	3.2 (1/31)	0 (0/61)	18.1 (2/11)	15.3 (2/13)	12.0 (3/25)	0.022 (χ^2^ test)
Improvement of penile rigidity *n* patients/total patients (see Fig. 1)	63.6 (7/11)	63.6 (14/22)	80.0 (4/5)	60.0 (3/5)	0 (0/9)	0.010 (χ^2^ test)
	Group 1 (°)	Group 2 (°)	Group 3 (°)	Group 4 (°)	Group 5 (°)	
						
Decrease of the penile curvature angle (average – degrees + standard deviation)	−9.6 ± 8.0	−14.0 ± 11.5	−2.7 ± 2.6	−4.5 ± 3.5	−3.9 ± 2.3	0.000 (anova)

In observing the plaque size reduction, excellent results in the case of hyperochoic plaques (−48.46%), good results in the case of plaque calicification (−30.54%) and poor results in the case of hypo and/or isoechoic plaques (−27.78%) were obtained. Concerning the reduction of penile curvature, similar results were observed: better results in the case of hyperochoic plaques (−7.6°), good results in the case of plaque calicification (−6.7°) and lower results in the case of hypo and/or isoechoic plaques (−6°).

Although in some categories of results, response rates were all high or not very different, the statistical study obviously could not detect a significant *p-value*. The significant finding, in fact, is objectively already evident in the value of the high response rate.

The worsening of Peyronie's disease (plaque size increase and/or increasing of curvature), despite treatment, occurred in 17 patients (17/151 = 11.2%). In only two cases, the increase in plaque size plus the increase in curvature occurred at the same time. In all these patients, the original size of the plaque exceeded 10 mm. When the plaque size increased, the volume increase was 10–40%. In all cases of curvature worsening, it increased from 5° to 45°.

The following risk factors were noted in 17 cases (15 patients) with worsening: a past history of radical retropubic prostatectomy for prostate cancer (one case); type 2 diabetes mellitus (two cases); a history of total proctocolectomy for ulcerative colitis (one case); presence of erectile dysfunction in seven cases (7/15 patients = 46.6% vs. 52/151 patients = 34.4%); penile plaques greater than 10 mm (15/15 patients = 100%); presence of multiple plaques (three patients); complex plaques in the form of armour (two patients); in 13 patients, the plaque was located in the distal part of the penis [ = 86.6% (13/15) vs. 29.8% (45/151 of total cases) (*p* = 0.00001 chi-square test)].

It is assumed that these results are mainly because of the presence of: erectile dysfunction; concomitant chronic diseases or debilitating conditions; large size of penile plaques; the location of plaques in the distal third of the penis. This last interesting item deals with a simple objective fact.

No cases of hypotension or heart rhythm disorder occurred.

## Discussion

This research has revealed that a ‘combination therapy’ was able to achieve greater results than each single drug alone (Lemourt Oliva *et al.*, 1998, 2003a, b, 2005; Riedl *et al.*, 2000; Levine & Estrada, 2002; Hauck *et al.*, 2006; Trost *et al.*, 2007; Taylor & Levine, 2008). In all groups and in most cases, a therapeutic response with regard to pain reduction, penile curvature, reduction of plaque size and improvement of penile rigidity (excluding group 5 with unsatisfactory results) was obtained. It should be noted, however, that the low mean effective reductions in plaque size were obtained in treatment groups 3, 4 and 5 (*p* = 0.000 anova); the low results obtained in these groups show that verapamil is more effective when a treatment programme includes both routes of administration (groups 1 and 2), while it also needs a contemporary treatment with vitamin E. Excellent response was obtained in reduction and disappearance of the plaque size in group 1, respectively *p* = 0.000 (anova) and *p* = 0.001 (chi-square test).

**Figure 1:**
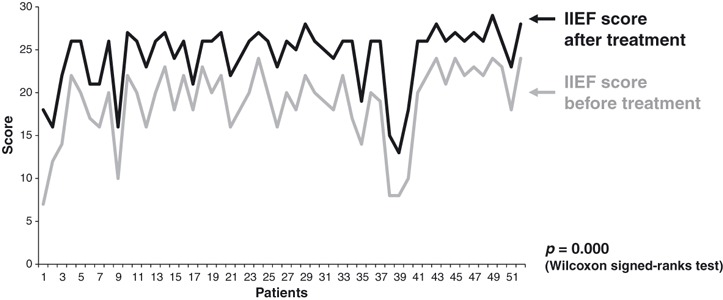
Erectile function in patients with Peyronie's disease (PD).

Excellent response was obtained in reduction and disappearance of the curvature in group 2 (propolis + others drugs), respectively, *p* = 0.000 (anova) and *p* = 0.019 (chi-square test). Good results regarding the reduction of the angle of penile curvature were also obtained in the first group. However, the unsatisfactory results related to group 5 (without vitamin E) in many *categories of results* show that vitamin E probably plays a significant role in the treatment of Peyronie's disease (contrary to what is reported by Hauck and other authors) especially if combined with other active drugs. The data for improved erectile function in all groups (see Fig. 1) excluding group 5 (*p* = 0.010 chi-square test) suggest that vitamin E may play a role in this situation.

When the plaque size increased, it was shown that the enlargement occurred more when verapamil was not administered in two ways. The worsening of the disease occurred more often in groups 3 and 4 (verapamil not administered in two ways) and 5 (without vitamin E), and it was statistically found to be significant (*p* = 0.02 chi-squared test) with regard to the progression of penile curvature. In only two cases, the plaque size enlargement was associated with the worsening of penile curvature. In the case of worsening of the penile curvature, note that in group 2 (combination of antioxidants vitamin E and propolis + verapamil in both modes of administration) there was no progression. Note that in contrast to common belief, penile calcifications may undergo a volume reduction or even disappear in response to conservative treatment. In fact, after treatment, most of penile calcifications were reduced in size (38/44 = 86.3%) (*p* = 0.022 chi-square test); also in this case, the best results were obtained when vitamin E was administered in combination with other drugs. These are interesting results considering that it is important to achieve the objective of reducing the amount of inextensible erectile tissue caused by calcification and/or fibrosis.

This study suggests that a ‘combination therapy’ for Peyronie's disease is able to achieve greater results than each drug administered separately. Verapamil is more effective when the treatment programme includes both routes of administration (peri-lesional injection + iontophoresis). The results of this retrospective study are encouraging and suggest that to increase the response to medical therapy, vitamin E should be combined with other drugs: verapamil (in both modes of administration), antioxidants and especially propolis and blueberries. Iontophoresis is a safe and non-invasive treatment for drug administration and the patient can autonomously perform this therapy; as home therapy is expected to be at least 6 months, the patient may directly buy or rent the appropriate equipment. Good results were achieved only after 6 months of treatment and this may explain why in other studies with the same drugs, but with a shorter time limit of the treatment, less satisfactory results were achieved. In case the option for conservative therapy exists, as Peyronie's disease is a chronic illness, short-term therapies should therefore be avoided.

For future reference, it is believed interesting to perform studies concerning the benefits of the use of PDE5 inhibitors in PD. In fact, recent studies show that these drugs can be effective in reversing the fibrosis of PD (Valente *et al.*, 2003; Ferrini *et al.*, 2006; Gonzalez-Cadavid & Rajfer, 2010) not only in rats but also in humans (Tadalafil) (Chung *et al.*, 2011).

Although this is only a retrospective study, however, it is important to report these results as they are to be considered interesting for good clinical procedures. To evaluate the efficacy of a conservative procedure for the treatment of Peyronie's disease, more prospective, randomized and placebo-controlled studies are needed. The various parameters of PD, especially penile curvature, plaque size and penile rigidity, should be evaluated by standardized instruments: penile dynamic ultrasound examinations, IIEF questionnaires, serial photographs of the curvature and pain scales.
